# Claims-based algorithms for common chronic conditions were efficiently constructed using machine learning methods

**DOI:** 10.1371/journal.pone.0254394

**Published:** 2021-09-27

**Authors:** Konan Hara, Yasuki Kobayashi, Jun Tomio, Yuki Ito, Thomas Svensson, Ryo Ikesu, Ung-il Chung, Akiko Kishi Svensson

**Affiliations:** 1 Department of Public Health, Graduate School of Medicine, The University of Tokyo, Bunkyo-ku, Tokyo, Japan; 2 Department of Economics, University of California, Berkeley, Berkeley, California, United States of America; 3 Precision Health, Department of Bioengineering, Graduate School of Engineering, The University of Tokyo, Bunkyo-ku, Tokyo, Japan; 4 Department of Clinical Sciences, Lund University, Skåne University Hospital, Malmö, Sweden; 5 School of Health Innovation, Kanagawa University of Human Services, Kawasaki-shi, Kanagawa, Japan; 6 Clinical Biotechnology, Center for Disease Biology and Integrative Medicine, Graduate School of Medicine, The University of Tokyo, Bunkyo-ku, Tokyo, Japan; 7 Department of Diabetes and Metabolic Diseases, Graduate School of Medicine, The University of Tokyo, Bunkyo-ku, Tokyo, Japan; Fuzhou University, CHINA

## Abstract

Identification of medical conditions using claims data is generally conducted with algorithms based on subject-matter knowledge. However, these claims-based algorithms (CBAs) are highly dependent on the knowledge level and not necessarily optimized for target conditions. We investigated whether machine learning methods can supplement researchers’ knowledge of target conditions in building CBAs. Retrospective cohort study using a claims database combined with annual health check-up results of employees’ health insurance programs for fiscal year 2016–17 in Japan (study population for hypertension, N = 631,289; diabetes, N = 152,368; dyslipidemia, N = 614,434). We constructed CBAs with logistic regression, k-nearest neighbor, support vector machine, penalized logistic regression, tree-based model, and neural network for identifying patients with three common chronic conditions: hypertension, diabetes, and dyslipidemia. We then compared their association measures using a completely hold-out test set (25% of the study population). Among the test cohorts of 157,822, 38,092, and 153,608 enrollees for hypertension, diabetes, and dyslipidemia, 25.4%, 8.4%, and 38.7% of them had a diagnosis of the corresponding condition. The areas under the receiver operating characteristic curve (AUCs) of the logistic regression with/without subject-matter knowledge about the target condition were .923/.921 for hypertension, .957/.938 for diabetes, and .739/.747 for dyslipidemia. The logistic lasso, logistic elastic-net, and tree-based methods yielded AUCs comparable to those of the logistic regression with subject-matter knowledge: .923-.931 for hypertension; .958-.966 for diabetes; .747-.773 for dyslipidemia. We found that machine learning methods can attain AUCs comparable to the conventional knowledge-based method in building CBAs.

## Introduction

A growing body of studies using medical and pharmacy claims data has been conducted in various fields of health research [1–7]. Among them, a notable amount of research has used claims data to assess medical conditions [1, 2, 6]. Despite its large volume of information and highly standardized format, however, claims data is frequently criticized for its potential imprecision in the identification of medical conditions mainly because they are primarily issued for reimbursement purpose [8–12].

To address these concerns, plenty of studies have proposed a claims-based algorithm (CBA) for identifying patients with their target condition and computed association measures to assess the usability of the algorithm [9, 10, 13–41]. Previous studies have engaged in a knowledge-based condition-specific CBA construction procedure, i.e., researchers selected input variables and decided how to incorporate them in the CBA based on their experience or existing clinical knowledge regarding the target condition. Although this approach is widely used and intuitively plausible, it is highly dependent on the level of knowledge on the target conditions and is hard to obtain appropriate and reproducible CBAs. This is imposing challenges to the use of administrative data in the transition from the ICD-9 to the ICD-10 coding scheme in the United States [42, 43].

Moreover, since previous CBA studies are predominantly coming from North American countries, research using diagnosis derived from North American countries’ claims data can be largely backed by a corresponding CBA study. In contrast, despite the rapid increase of research using diagnosis derived from claims data in other countries–e.g., Japan and Taiwan–CBAs are not established for most medical conditions thus far [44]. It is notable that the lack of confirmed CBA not only degrades the quality of research but also makes the research extremely difficult to be accepted by journals with high impact factors [45]. For this reason, researchers who are using claims data in these countries are facing an urgent need to establish CBAs for various medical conditions.

To this end, some researchers applied conventional regression methods to develop CBAs which are less dependent on the knowledge [9, 14, 17, 19, 24, 35, 37, 40]. However, the selection of input variables is required before implementing a regression model to obtain a satisfactory CBA, as conventional regression methods often work poorly in prediction accuracy when the number of input variables is large relative to the sample size [46]. Besides, if researchers expect nonlinear or interactive effects of the input variables, they have to specify those terms *a priori* as a functional form of the regression model.

Machine learning methods are promising technologies to overcome the problems of conventional regression methods, and some researchers have attempted to use these methods in the context of CBA [18, 25, 29, 30, 39]. However, they selected the input variables according to their target condition. Thus, to apply their procedures to other conditions, it is necessary to start over from the variable selection. Additionally, among different methods for machine learning, the methods better suited than others for developing CBAs have not been addressed yet.

In this study, using a large database of employees’ health insurance programs, we developed CBAs with selected machine learning methods for identifying patients with three common chronic conditions: hypertension, diabetes, and dyslipidemia. We then compared their association measures using a hold-out test set.

## Methods

### Institutional settings

The Japanese government provides a universal health insurance program for all registered inhabitants. Besides, each employer is obliged by law to provide annual health check-up to its employees. Medical and pharmacy claims data combined with annual health check-up results of employees’ health insurance programs were obtained in an anonymous format from JMDC Inc. [47]. Further details on the institutional settings have been described previously [33].

Claims data contain enrollee information, including gender, month and year of birth, and their diagnostic code, medical institutions, pharmacies, and medical treatments provided. Diagnostic and medication codes are classified by the 2003 version of the International Statistical Classification of Diseases and Related Health Problems, Tenth Revision (ICD-10) [48] and 2016 version of the World Health Organization-Anatomical Therapeutic Chemical (WHO-ATC) [49], respectively. Enrollees’ age was defined as their age in March 2018. Annual health check-up results include information on the results of the physical examination and blood test, whether fasting blood samples were collected, and the answer to a health-related questionnaire including questions on medication usage. The study protocol was approved by the research ethics committee of the University of Tokyo (approval number: KE18-44). The ethics committee waived informed consent because this is a retrospective study with the data that were fully anonymized before we accessed them. JMDC Inc. applies strict policies to protect the privacy of enrollees and medical providers, and all private information that could identify enrollees and medical providers were removed beforehand [47].

### Study population

**Fig 1:**
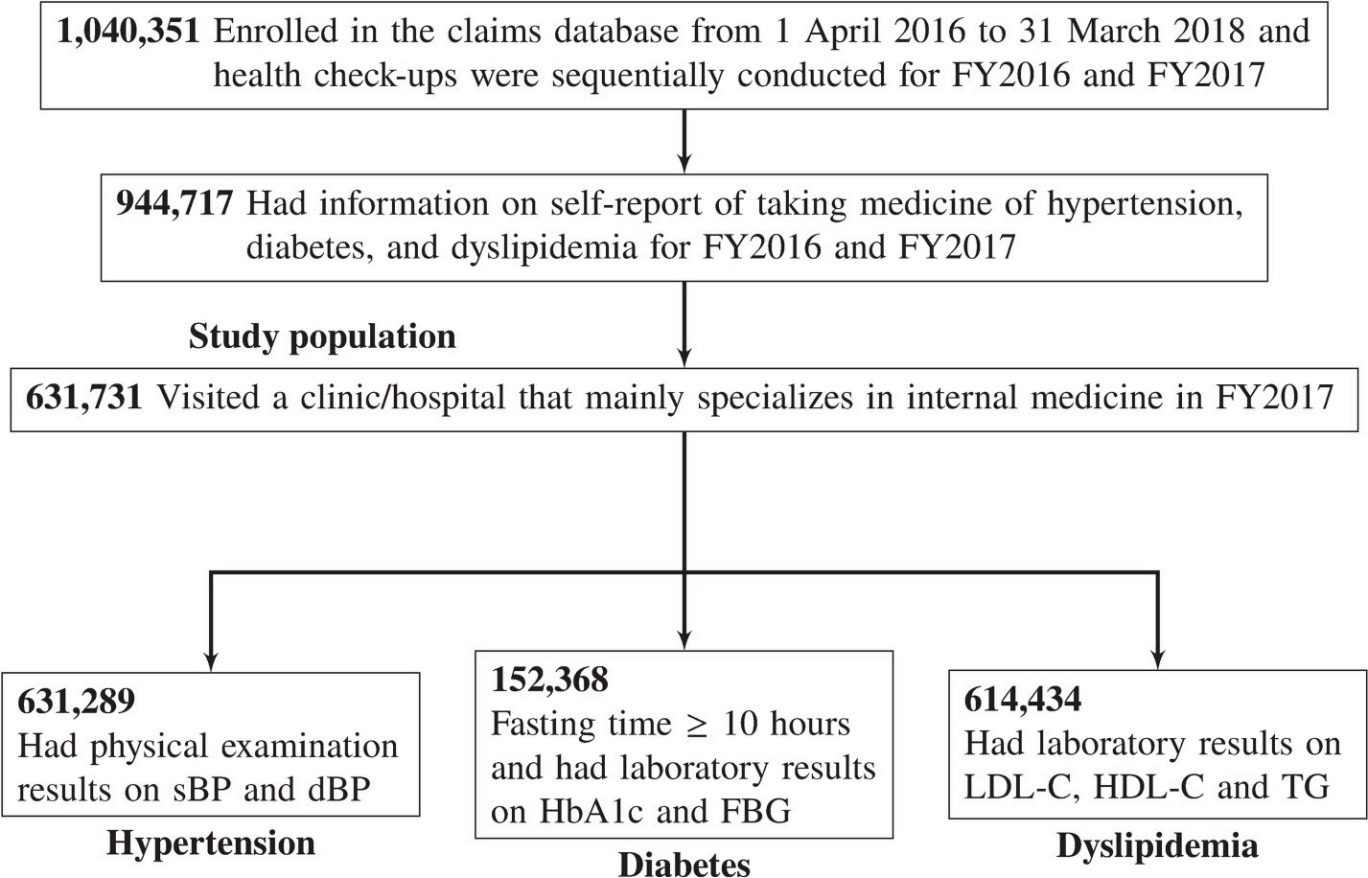
Flowchart of inclusion and exclusion of study participants. *Abbreviations*: dBP, diastolic blood pressure; FBG, fasting blood glucose; FY, fiscal year; HbA1c, hemoglobin A1c; HDL-C, high-density lipoprotein cholesterol; LDL-C, low-density lipoprotein cholesterol; sBP, systolic blood pressure; TG, triglyceride.

The study population for each condition of hypertension, diabetes, and dyslipidemia was defined as beneficiaries (1) who were enrolled in the claims database from April 1, 2016, to March 31, 2018, and whose health check-ups were sequentially conducted for fiscal year (FY)2016 and FY2017 (N = 1,040,351), (2) with complete data on the self-reported use of blood pressure- and lipid-lowering drugs and hypoglycemic drugs for FY2016 and FY2017 (N = 944,717), (3) who in FY2017 visited a clinic/hospital that mainly specializes in internal medicine (N = 631,731), and (4) with complete data on examination results required for the gold standard of each condition mentioned later for FY2016 and FY2017 (hypertension, N = 631,289; diabetes, N = 152,368; dyslipidemia, N = 614,434) (Fig 1).

In similar studies to date, chart review has often been the source of the gold standard, with the population to calculate association measures constrained to those who visited primary care facilities [15, 16, 19]. To make the present study comparable to the past research, we restricted the study population to those who, at least once in the FY, had visited a clinic/hospital that mainly specializes in internal medicine, which has the function of primary care in Japan.

### Gold standard and claims-based algorithm

We constructed a gold standard to diagnose each condition from the health check-up results of FY2016 and FY2017 as previously described (Table 1) [33]. We used FY2017 claims data as the source of the CBA and compared it with the diagnosis derived from the gold standard. The scheme of using one-year claims data corresponding to the latter health check-up year for developing CBAs is the same as that of the previous study [33].

**Table 1:** Gold standards to diagnose hypertension, diabetes, and dyslipidemia.

Diagnose as hypertension if any of the following conditions are satisfied: 1. Systolic blood pressure ≥ 140 mmHg and/or diastolic blood pressure ≥ 90 mmHg for FY2016 and FY2017 2. Self-report of taking blood pressure-lowering drugs in at least one of FY2016 and FY2017*
Diagnose as diabetes if any of the following conditions are satisfied: 1. HbA1c ≥ 6.5% in at least one of the two years and FBG ≥ 126 mg/dL in at least one of FY2016 and FY2017 2. FBG ≥ 126 mg/dL for FY2016 and FY2017 3. Self-report of taking hypoglycemic drugs in at least one of FY2016 and FY2017*
Diagnose as dyslipidemia if any of the following conditions are satisfied: 1. Low-density lipoprotein cholesterol ≥ 140 mg/dL for FY2016 and FY2017 2. High-density lipoprotein cholesterol ≤ 40 mg/dL for FY2016 and FY2017 3. Triglyceride ≥ 150 mg/dL for FY2016 and FY2017 4. Self-report of taking lipid-lowering drugs in at least one of FY2016 and FY2017*

*Abbreviations*: HbA1c, hemoglobin A1c; FBG, fasting blood glucose; FY, fiscal year.

*The reliability of the self-report of medication usage was demonstrated to be satisfactorily high when compared with the pharmacy claims-based drug usage [33].

To construct CBAs, we first set up a dataset containing the input variables that can be chosen without subject-matter knowledge on the target conditions, namely, age, gender, and the number of observations of each of ICD-10/WHO-ATC code with a letter followed by two digits (main dataset). We counted the observations of ICD-10/WHO-ATC codes on claims as one occurrence when the information was accrued from the same month. We excluded the ICD-10 codes for suspected cases and counted the ICD-10 codes regardless of whether they were listed as primary diagnoses. We then applied following popular machine learning methods, (1) k-nearest neighbor (kNN), (2) support vector machine (SVM), (3) penalized logistic regression, (4) tree-based model, and (5) neural network, to the dataset.

Additionally, as benchmarks, we developed two sets of conventional CBAs. Firstly, we emulated two manually constructed CBAs proposed in the previous study [33]. Patients meeting the following selection rule were classified as “test-positive” for condition X (hypertension, diabetes, or dyslipidemia): (1) the diagnostic code corresponding to condition X is found in the claims at least once (diagnostic code-based CBA); and (2) the medication code corresponding to condition X is found in the claims at least once (medication code-based CBA).

Secondly, we applied a logistic regression model to the main dataset and an alternative dataset where input variables were selected according to each condition. The logistic regression model with the alternative dataset corresponds to a typical procedure among the conventional knowledge-based methods in building CBAs. The selected input variables were age, gender, and the number of observations of each of ICD-10/WHO-ATC codes that corresponds to the target condition. The ICD-10 codes corresponding to hypertension, diabetes, and dyslipidemia were defined as I10-I15, E10-E14, and E78, respectively. The WHO-ATC codes corresponding to hypertension, diabetes, and dyslipidemia were defined as C08 and/or C09, A10, and C10, respectively.

### Association measures

We quantified the goodness of CBAs by the following association measures: sensitivity, specificity, positive predictive value (PPV), negative predictive value (NPV), receiver operating characteristic (ROC) curve, and area under the ROC curve (AUC). For the calculation of the association measures, true positive and test positive cases were defined as the enrollees who were assessed as having a disease by the gold standard and those who were identified as having a disease by the CBA, respectively.

### Statistical analysis

We randomly divided the dataset into two sets: training (75%), which was used to estimate parameters and tune hyperparameters; test (25%), which was used to assess the association measures of the CBA. The sensitivity, specificity, PPV, and NPV were estimated for the diagnostic code- and medication code-based CBAs, and their 95% confidence intervals (CIs) were calculated using exact binomial confidence limits [50]. We calculated these association measures and 95% CIs using the *epiR* package [51].

We estimated a prediction function that outputs the score of the propensity for having a disease given a set of input variables using the selected methods. The outcome variable in hand is a binary indicator of having a disease that is assessed by the gold standard. For each selected machine learning method, we chose several types of prediction procedures that are commonly applied. The Euclidean distance with raw or standardized (i.e., rescaled to have mean zero and variance one) input variables was adopted as a distance metric for the kNN [52, 53]. A linear basis function with a hinge or squared hinge loss was adopted in the SVM [54]. From the penalized logistic regression, logistic regressions with the *L*_2_-penalty (logistic ridge) [55], *L*_1_-penalty (logistic lasso) [56], and elastic-net penalty (logistic elastic-net) [57] were applied. Two types of tree-based models were applied: random forest [58] and importance sampled learning ensemble (ISLE) [59]. A single hidden layer neural network was applied with a different number of hidden units: 5, 10, and 20 [60].

If the model involved a hyperparameter to be tuned, the training set was used for the tuning. The expected value of the AUC was estimated through tenfold cross-validation with the training set. If the computational burden of tenfold cross-validation was prohibitive, we used a validation set to estimate the expected value of the AUC. A third of the training set was chosen at random to construct the validation set. Which of tenfold cross-validation or a validation set was used for each model is described below. The hyperparameter was then chosen to be the value that maximized the AUC. After the hyperparameter determination, the training set was used again to estimate parameters for the prediction function. When no hyperparameter tuning was required, the training set was used to estimate parameters in the prediction function from the beginning. We described the details of the parameter estimation and hyperparameter tuning for each method in the following.

#### Logistic regression

The outcome variable was regressed on the input variables to generate a prediction function. The analysis of the logistic regression was implemented by the *mnlogit* package [61].

#### k-nearest neighbor

The number of the nearest neighbors to be counted, k, was optimized using the validation set. The predicted class probabilities that were computed from (1) the frequency of the class of the k-nearest neighbors (vote) [52] and (2) the inverse distance weighted frequency of the class of the k-nearest neighbors (IDW) [53] composed a prediction function. The analysis of the kNN was implemented by the *fastknn* package [62].

#### Support vector machine

The cost parameter was optimized using the validation set. Decision values (i.e., the distance of the point from the hyperplane) made up a prediction function. The analysis of the SVM was implemented by the *LiblineaR* package [63].

#### Penalized logistic regression

The regularization coefficient and elastic-net mixing parameter were determined by cross-validation. The analysis of the penalized logistic regression model was implemented by the *glmnet* package [64].

#### Tree-based model

The minimum node size was set to 10 for each tree, and 200 trees were bagged in the random forest. The number of variables selected for each split was tuned using the validation set. The probability forest was used to generate a prediction function [65]. The analysis of the random forest was implemented by the *ranger* package [66]. There are five hyperparameters in the importance sampled learning ensemble (ISLE): a hyperparameter for the tree size, subsampling ratio for each tree, learning rate, number of trees to be bagged, and regularization coefficient for the post-processing. We adopted the depth of the tree as the hyperparameter for the tree size and fixed it to be six [60]. As the combination of the subsampling ratio for each tree and learning rate, we selected (1,0.05), (0.5,0.1), and (0.1,0.1). Since the basis function generating process of the ISLE is identical to that of the gradient boosting machine (GBM) if the subsampling ratio is one and that of the stochastic gradient boosting machine (SGBM) if otherwise, we set the learning rate according to Friedman’s recommendation for the GBM and SGBM [67, 68]. The remaining two hyperparameters, the number of trees to be bagged and regularization coefficient, were determined by cross-validation. In particular, for a given value of the regularization coefficient, the basis function generating process was stopped if the cross-validation AUC did not improve for three basis function generating rounds. The value with the maximum cross-validation AUC was then chosen as the regularization coefficient for the prediction function. The *L*_1_-penalty was adopted in the post-processing following the recommendation of Friedman and Popescu (2003) [59]. The analysis of the ISLE was implemented by the *xgboost* package [69].

#### Neural network

All hidden units were fully connected with the nodes in the input and output layers. Weight decay was employed for the regularization of parameters, and the regularization coefficient of it was tuned using the validation set. The analysis of the neural network was implemented by the *nnet* package [70].

Provided an estimated prediction function from the model, an ROC curve was drawn from the scores and the matched observed outcome values as the threshold of considering a patient positive were moved over the range of all possible scores. The AUC was calculated from the resulting ROC curve, and DeLong’s method was used to determine the 95% CI for the AUC [71].

In the end, a representative point of sensitivity and specificity on the ROC curve was chosen based on the Youden index [72, 73]. The PPV and NPV were calculated according to the representative point. Moreover, the 95% CIs for the sensitivity, specificity, PPV, and NPV were calculated with 200 bootstrap resampling and the averaging methods as described previously [74]. We drew the ROC curve and calculated the association measures and their 95% CIs using the *pROC* package [75]. All statistical analysis was conducted using R version 3.6.1 [76]. R codes are available at https://github.com/harakonan/research-public/tree/master/cba.

## Results

### Summary statistics

Table 2 tabulates the summary statistics of 944,717 enrollees’ characteristics and health check-up results for each fiscal year. The mean age was 48.0 years (standard deviation ± 10.4 years). More than 80% of people received fasting blood tests. Furthermore, 85% of the enrollees visited any clinics/hospitals during the year, while 67% went to the primary care clinics/hospitals. Among the test cohorts of 157,822, 38,092, and 153,608 enrollees for hypertension, diabetes, and dyslipidemia, 25.4%, 8.4%, and 38.7% of them had a diagnosis of the corresponding condition.

**Table 2:** Summary statistics of enrollees’ characteristics and health check-up results for each fiscal year of the enrollees with complete data on the self-reported use of blood pressure- and lipid-lowering drugs and hypoglycemic drugs (N = 944,717).

	FY2016			FY2017		
Variables	Mean	SD	Missing (%)	Mean	SD	Missing (%)
Demographics						
Male	−	−	−	0.8	−	−
Age* (year)	−	−	−	48	10.4	−
Visited clinic/hospital						
Any clinic/hospital^†^	0.85	−	−	0.85	−	−
Primary care clinic/hospital^‡^	0.67	−	−	0.67	−	−
Health check-up results						
Fasting time ≥ 10 hours^§^	0.81	−	54.9	0.81	−	56.8
Systolic blood pressure (mmHg)	121.5	15.8	0.1	122.1	15.9	0.0
Diastolic blood pressure (mmHg)	75.5	11.7	0.1	75.9	11.8	0.0
Fasting blood glucose (mg/dL)	96.7	18.5	20.5	97.3	19	21.1
Hemoglobin A1c (%)	5.56	0.64	15.7	5.59	0.64	14.5
Low-density lipoprotein cholesterol (mg/dL)	121.1	30.8	2.4	121.3	30.6	2.8
High-density lipoprotein cholesterol (mg/dL)	60.6	15.9	2.4	60.9	16.1	2.8
Triglyceride (mg/dL)	117.1	94	2.4	118.3	94.5	2.8
Self-report of taking drug^¶^						
Blood-pressure-lowering drugs	0.12	−	−	0.13	−	−
Hypoglycemic drugs	0.04	−	−	0.04	−	−
Lipid-lowering drugs	0.07	−	−	0.08	−	−

*Abbreviations*: FY, fiscal year; SD, standard deviation.

Notes: Only mean (or proportion) is stated for a categorical variable. Because the variables “Male” and “Age” do not change with the year, we only tabulated them in column FY2017. There are no missing values in the variables other than the health check-up results by construction.

*Age is defined as the age in March 2018.

^†^Any clinic/hospital indicates that a person visited any kind of clinic/hospital in the corresponding FY.

^‡^Primary care clinic/hospital indicates that a person visited a clinic/hospital that mainly provides internal medicine in the corresponding FY.

^§^Fasting time ≥ 10 hours indicates if more than 10 hours have passed since the last meal when blood samples were collected.

^¶^Self-report of taking drugs are extracted from the answer to a health-related questionnaire.

Table 3 displays the cumulative counts and distribution of the proportion of enrollees whose claims contain the ICD-10/WHO-ATC code at least once in the study population. The numbers of the ICD-10 and WHO-ATC codes that appeared in the dataset for the study population were 1333 and 92, respectively. Nearly 90% of the ICD-10 codes that appeared in the dataset were only observed for less than 1% of enrollees, and more than half of the WHO-ATC codes that appeared in the dataset were observed for less than 5% of enrollees.

**Table 3:** Cumulative distribution of the proportion of enrollees whose claims contain the ICD-10/WHO-ATC code at least once in the study population (N = 631,731).

	ICD-10 code		WHO-ATC code	
Proportion	Count	Percentile	Count	Percentile
≤ 0.01%	485	36.4 th	5	5.4 th
≤ 0.1%	879	65.9 th	12	13.0 th
≤ 1%	1195	89.6 th	32	34.8 th
≤ 2%	1254	94.1 th	39	42.4 th
≤ 3%	1277	95.8 th	45	48.9 th
≤ 5%	1302	97.7 th	49	53.3 th
≤ 10%	1318	98.9 th	69	75.0 th
≤ 20%	1326	99.5 th	80	87.0 th
≤ 30%	1331	99.8 th	86	93.5 th
≤ 50%	1333	100.0 th	91	98.9 th
≤ 100%	1333	100.0 th	92	100.0 th

*Abbreviations*: ICD-10, International Classification of Diseases and Related Health Problems, tenth revision; WHO-ATC, World Health Organization-anatomical therapeutic chemical.

Notes: For each two-digit ICD-10/WHO-ATC code, the proportion of enrollees whose claims contain the code at least once was computed for the study population. Cumulative counts and distribution of the computed proportion was tabulated separately for ICD-10 codes and WHO-ATC codes. The count (percentile) column tabulates the number (fraction) of two-digit ICD-10/WHO-ATC codes that the proportion of enrollees whose claims contain the code at least once is below the value in the proportion column.

### Association measures

Table 4 reports the association measures and their 95% CIs for the diagnostic code- and medication code-based CBAs. The sensitivity, specificity, PPV, and NPV closely followed those values computed previously [33]. The diagnostic code-based CBAs had higher sensitivity and NPV but lower specificity and PPV compared to the medication code-based CBAs. For hypertension, all association measures were acceptably high, while, for diabetes, the diagnostic code-based CBA fell short of a satisfactory level of the PPV. For dyslipidemia, the sensitivity of both CBAs was considerably lower than those for hypertension and diabetes.

**Table 4:** Association measures and their 95% confidence intervals for the diagnostic code- and medication code-based claims-based algorithms.

	Sensitivity			Specificity			PPV			NPV		
Method	%	95%CI		%	95%CI		%	95%CI		%	95%CI	
Hypertension (N = 157,822, Prevalence = 25.4%)												
Diagnostic code	80.7	80.3	81.1	95.2	95.0	95.3	85.0	84.6	85.4	93.6	93.4	93.7
Medication code	75.3	74.9	75.7	97.8	97.7	97.9	92.0	91.7	92.3	92.1	91.9	92.2
Diabetes (N = 38,092, Prevalence = 8.4%)												
Diagnostic code	90.8	89.8	91.8	92.9	92.6	93.2	53.8	52.5	55.2	99.1	99.0	99.2
Medication code	79.3	77.8	80.6	99.5	99.4	99.6	93.5	92.5	94.4	98.1	98.0	98.3
Dyslipidemia (N = 153,608, Prevalence = 38.7%)												
Diagnostic code	49.6	49.2	50.0	90.0	89.8	90.2	75.9	75.5	76.3	73.9	73.6	74.1
Medication code	36.2	35.8	36.6	96.9	96.8	97.0	88.1	87.7	88.5	70.6	70.3	70.8

*Abbreviations*: CI, confidence interval; NPV, negative predictive value; PPV, positive predictive value.

Patients meeting the following selection rule were classified as “test-positive” for each condition: (1) the diagnostic code corresponding to the condition is found in the claims at least once (diagnostic code-based claims-based algorithm); and (2) the medication code corresponding to the condition is found in the claims at least once (medication code-based claims-based algorithm). We calculated 95% CIs for all estimates of sensitivity, specificity, PPV, and NPV using exact binomial confidence limits.

Table 5 shows the association measures and their 95% CIs for the CBAs derived from the machine learning methods for hypertension (Table 5A), diabetes (Table 5B), and dyslipidemia (Table 5C). ROC curves are shown in S1 File.

**Table 5:** Association measures and their 95% confidence intervals for claims-based algorithms derived from machine learning methods.

**A. Hypertension (N = 157,822, Prevalence = 25.4%)**															
	AUC			Sensitivity			Specificity			PPV			NPV		
Method		95%CI		%	95%CI		%	95%CI		%	95%CI		%	95%CI	
Logistic regression															
Main dataset	0.921	0.919	0.923	78.5	78.0	79.1	95.7	95.3	96.1	86.0	85.1	87.1	92.9	92.8	93.1
Alternative dataset	0.923	0.921	0.924	78.0	77.5	78.6	96.1	95.6	96.3	87.0	85.7	87.8	92.8	92.6	92.9
k-nearest neighbor															
Vote	0.917	0.915	0.919	77.2	76.8	77.9	96.1	95.4	96.3	87.2	85.3	87.5	92.6	92.4	92.7
Vote-Standardized	0.847	0.844	0.849	72.7	70.7	73.1	81.8	81.6	83.6	57.6	57.2	59.5	89.8	89.4	90.0
IDW	0.915	0.913	0.917	77.3	76.8	78.0	95.9	95.4	96.2	86.5	85.3	87.3	92.6	92.4	92.7
IDW-Standardized	0.845	0.842	0.847	72.4	70.6	73.5	81.9	80.8	83.6	57.6	56.6	59.5	89.7	89.3	90.0
Support vector machine															
Hinge loss	0.916	0.914	0.918	76.9	76.4	77.7	95.5	94.7	95.8	85.2	83.3	86.2	92.4	92.3	92.6
Squared hinge loss	0.923	0.921	0.924	79.6	79.3	80.2	95.7	95.4	95.9	86.2	85.5	86.8	93.3	93.1	93.4
Penalized logistic regression															
Logistic Ridge	0.892	0.890	0.894	77.1	76.3	77.8	88.0	87.6	88.7	68.7	67.9	69.7	91.9	91.7	92.1
Logistic Lasso	0.924	0.922	0.925	78.7	78.2	79.1	95.6	95.3	96.1	86.0	85.2	87.4	93.0	92.8	93.1
Logistic Elastic-net	0.923	0.921	0.925	78.7	78.2	79.1	95.5	95.2	95.7	85.6	84.8	86.2	92.9	92.8	93.1
Tree-based method															
Random Forest	0.923	0.922	0.925	80.5	79.8	81.1	95.5	95.2	96.1	85.8	85.0	87.5	93.5	93.3	93.7
ISLE-sample 1 learn 0.05	0.931	0.929	0.932	80.5	80.1	81.0	95.8	95.5	96.0	86.8	85.9	87.3	93.5	93.4	93.7
ISLE-sample 0.5 learn 0.1	0.931	0.929	0.932	80.7	80.2	81.1	95.7	95.3	95.9	86.3	85.4	87.0	93.6	93.4	93.7
ISLE-sample 0.1 learn 0.1	0.930	0.928	0.932	80.7	80.2	81.2	95.6	95.2	95.9	86.1	85.2	86.8	93.6	93.4	93.7
Neural network															
Hidden units 5	0.918	0.916	0.919	79.6	78.9	80.2	94.9	94.5	95.4	84.2	83.1	85.4	93.2	93.0	93.4
Hidden units 10	0.922	0.920	0.924	79.4	78.8	80.1	95.6	94.9	96.0	85.9	84.3	87.1	93.2	93.0	93.4
Hidden units 20	0.921	0.919	0.923	79.3	78.8	79.9	95.3	94.7	95.6	85.1	83.6	85.9	93.1	93.0	93.3
**B. Diabetes (N = 38,092, Prevalence = 8.4%)**															
	AUC			Sensitivity			Specificity			PPV			NPV		
Method		95%CI		%	95%CI		%	95%CI		%	95%CI		%	95%CI	
Logistic regression															
Main dataset	0.938	0.932	0.944	85.0	83.4	86.4	95.4	94.1	96.4	62.6	56.8	67.8	98.6	98.5	98.7
Alternative dataset	0.957	0.952	0.961	86.9	85.8	88.4	95.6	94.7	95.9	64.3	59.9	65.9	98.8	98.7	98.9
k-nearest neighbor															
Vote	0.942	0.936	0.948	84.5	82.9	86.2	94.8	93.5	95.8	59.8	54.2	64.2	98.5	98.4	98.7
Vote-Standardized	0.884	0.877	0.891	77.6	75.1	81.9	84.7	80.4	87.3	31.5	27.5	34.7	97.6	97.4	98.0
IDW	0.942	0.936	0.948	84.7	82.9	86.9	95.0	92.9	96.0	60.7	52.5	65.5	98.6	98.4	98.7
IDW-Standardized	0.885	0.878	0.892	78.8	75.8	82.9	83.6	79.8	86.0	30.5	27.0	33.3	97.7	97.5	98.1
Support vector machine															
Hinge loss	0.944	0.938	0.950	86.0	84.3	87.7	95.4	94.0	96.5	62.9	57.0	68.7	98.7	98.5	98.8
Squared hinge loss	0.945	0.939	0.951	86.9	84.9	88.1	95.7	95.0	96.9	64.8	61.5	71.6	98.8	98.6	98.9
Penalized logistic regression															
Logistic Ridge	0.928	0.922	0.933	83.3	80.5	85.1	89.7	88.0	91.8	42.3	39.0	47.2	98.3	98.1	98.5
Logistic Lasso	0.959	0.955	0.964	88.7	87.4	89.8	94.8	94.3	95.4	60.8	58.5	63.5	98.9	98.8	99.0
Logistic Elastic-net	0.960	0.956	0.964	88.9	87.8	89.9	94.5	93.9	95.0	59.7	57.0	61.5	98.9	98.8	99.0
Tree-based method															
Random Forest	0.958	0.953	0.962	88.5	86.5	90.0	94.9	93.5	96.6	61.3	55.8	70.0	98.9	98.7	99.0
ISLE-sample 1 learn 0.05	0.966	0.962	0.970	89.8	88.5	90.9	94.7	93.8	95.1	60.5	56.9	62.4	99.0	98.9	99.1
ISLE-sample 0.5 learn 0.1	0.965	0.961	0.969	89.8	88.7	91.2	94.5	93.5	95.0	59.9	56.2	61.9	99.0	98.9	99.2
ISLE-sample 0.1 learn 0.1	0.964	0.960	0.968	90.3	88.3	91.4	93.7	92.9	95.4	56.5	53.9	64.1	99.1	98.9	99.2
Neural network															
Hidden units 5	0.938	0.932	0.944	83.3	81.2	85.0	94.5	93.1	96.1	57.9	52.6	65.8	98.4	98.2	98.6
Hidden units 10	0.940	0.935	0.946	83.7	81.9	86.0	95.4	93.3	96.4	62.5	53.6	67.7	98.5	98.3	98.6
Hidden units 20	0.938	0.932	0.944	83.9	82.3	85.8	95.8	94.2	97.0	64.7	57.1	71.5	98.5	98.3	98.6
**C. Dyslipidemia (N = 153,608, Prevalence = 38.7%)**															
	AUC			Sensitivity			Specificity			PPV			NPV		
Method		95%CI		%	95%CI		%	95%CI		%	95%CI		%	95%CI	
Logistic regression															
Main dataset	0.747	0.744	0.749	48.8	43.8	51.1	86.1	83.9	91.0	69.2	66.7	75.5	72.6	71.9	73.1
Alternative dataset	0.739	0.736	0.742	42.6	42.1	43.4	91.8	91.1	92.1	76.6	75.3	77.2	71.6	71.5	71.8
k-nearest neighbor															
Vote	0.742	0.739	0.745	46.6	45.1	49.5	89.3	86.4	90.4	73.2	69.5	75.0	72.6	72.3	73.0
Vote-Standardized	0.678	0.676	0.681	54.6	47.6	55.4	69.5	69.0	76.4	53.2	52.7	56.1	70.7	69.7	71.0
IDW	0.740	0.737	0.742	48.2	45.3	50.0	87.3	85.7	90.4	70.9	69.0	74.7	72.7	72.3	73.0
IDW-Standardized	0.675	0.673	0.678	53.8	49.3	54.9	70.3	69.3	74.6	53.4	52.9	55.3	70.6	69.9	70.9
Support vector machine															
Hinge loss	0.738	0.735	0.740	49.6	49.0	50.0	90.0	89.8	90.2	75.8	75.4	76.2	73.8	73.6	74.0
Squared hinge loss	0.748	0.746	0.751	50.0	45.7	50.6	85.2	84.9	89.3	68.2	67.7	73.0	72.9	72.2	73.1
Penalized logistic regression															
Logistic Ridge	0.726	0.723	0.728	54.8	51.2	58.2	76.6	73.2	80.2	59.8	57.9	62.0	72.8	72.2	73.5
Logistic Lasso	0.753	0.751	0.756	49.3	48.9	49.7	90.3	90.0	90.5	76.3	75.8	76.7	73.8	73.6	74.0
Logistic Elastic-net	0.747	0.744	0.749	45.5	44.5	47.4	89.7	87.7	90.4	73.5	70.8	74.7	72.2	72.0	72.6
Tree-based method															
Random Forest	0.763	0.761	0.766	49.8	48.3	50.3	90.0	89.7	91.4	76.0	75.4	78.0	73.9	73.6	74.1
ISLE-sample 1 learn 0.05	0.773	0.771	0.775	51.2	49.9	52.0	88.9	88.0	90.1	74.5	73.2	76.2	74.2	74.0	74.4
ISLE-sample 0.5 learn 0.1	0.773	0.770	0.775	51.4	49.5	52.5	88.8	87.5	90.6	74.3	72.7	76.9	74.2	73.9	74.5
ISLE-sample 0.1 learn 0.1	0.771	0.768	0.773	50.0	49.0	51.5	90.0	88.4	90.9	75.9	73.6	77.4	74.0	73.8	74.3
Neural network															
Hidden units 5	0.748	0.745	0.750	48.3	46.6	50.8	87.7	85.3	89.4	71.3	68.5	73.5	72.9	72.5	73.3
Hidden units 10	0.751	0.749	0.754	47.7	46.7	50.0	89.3	86.8	90.2	73.9	70.6	75.2	73.0	72.8	73.3
Hidden units 20	0.758	0.755	0.760	49.4	48.3	51.8	88.5	86.0	89.5	73.0	70.1	74.5	73.5	73.2	73.8

*Abbreviations*: AUC, area under the receiver operating characteristic curve; CI, confidence interval; IDW, inverse distance weighting; ISLE, importance sampled learning ensemble; NPV, negative predictive value; PPV, positive predictive value.

Notes: Age, gender, and all International Classification of Diseases and Related Health Problems, Tenth Revision (ICD-10)/World Health Organization-Anatomical Therapeutic Chemical (WHO-ATC) codes with a letter followed by two digits were used as input variables for all models but the logistic regression using the alternative dataset. The main logistic regression fitted a logistic regression model to the dataset that was appropriately trimmed. The Euclidean distance with raw or standardized (i.e., rescaled to have mean zero and variance one) input variables was adopted as a distance metric for the k-nearest neighbor (kNN). The number of the nearest neighbors to be counted, k, was optimized using the validation set. The predicted class probabilities that were computed from (1) the frequency of the class of the k-nearest neighbors (vote) and (2) the inverse distance weighted frequency of the class of the k-nearest neighbors (IDW) composed a prediction function. A linear basis function with a hinge or squared hinge loss was adopted in the support vector machine (SVM). The cost parameter was optimized using the validation set. Decision values (i.e., the distance of the point from the hyperplane) made up a prediction function. From the penalized logistic regression, logistic regression with the *L*_2_-penalty (logistic ridge), *L*_1_-penalty (logistic lasso), and elastic-net penalty (logistic elastic-net) were applied. The regularization coefficient and elastic-net mixing parameter were determined by cross-validation. Two types of tree-based models were applied: random forest and importance sampled learning ensemble (ISLE). The minimum node size was set to 10 for each tree, and 200 trees were bagged in the random forest. The number of variables selected for each split was tuned using the validation set. We fixed the depth to be six for the ISLEs. As the combination of the subsampling ratio for each tree and the learning rate, we selected (1,0.05), (0.5,0.1), and (0.1,0.1). The number of trees and regularization coefficient were determined by cross-validation. The *L*_1_-penalty was adopted in the post-processing. A single hidden layer neural network was applied with a different number of hidden units: 5, 10, and 20. All hidden units were fully connected with the nodes in the input and output layers. Weight decay was employed for the regularization of parameters, and the regularization coefficient of it was tuned using the validation set. Delong’s method was used to determine the 95% CI for the AUC. A representative point of sensitivity and specificity on the ROC curve is chosen based on the Youden index. PPV and NPV were calculated according to the representative point, and the 95% CIs for the resulting sensitivity, specificity, PPV, and NPV were calculated with 200 bootstrap resampling and the averaging methods.

The AUC of the logistic regression with subject-matter knowledge about the target condition, i.e., the logistic regression with the alternative dataset, was .923 for hypertension, .957 for diabetes, and .739 for dyslipidemia. The representative sensitivity, specificity, PPV, and NPV of this method were comparable to those of the convex combination of the diagnostic code- and medication code-based CBAs: hypertension, sensitivity 78.0%, specificity 96.1%, PPV 87.0%, and NPV 92.8%; diabetes, 86.9%, 95.6%, 64.3%, and 98.8%; dyslipidemia, 42.6%, 91.8%, 76.6%, and 71.6%. Without subject-matter knowledge about the target condition, i.e., the logistic regression with the main dataset, the AUC for hypertension stayed similar, .921, that for diabetes decreased to .938, and that for dyslipidemia increased to .747.

The logistic lasso, logistic elastic-net, and tree-based methods yielded AUCs that were comparable to or higher than those of the logistic regression with subject-matter knowledge: logistic lasso, .924 for hypertension, .959 for diabetes, and .753 for dyslipidemia; logistic elastic-net, .923, .960, and .747; random forest, .923, .958, and .763; ISLE (the range from three hyperparameter specifications), .930–.931, .964–.966, and .771–.773.

The kNN with raw input variables, SVM, and neural network attained AUCs that were comparable to those of the logistic regression without subject-matter knowledge: kNN with raw input variables (the range from the vote and IDW), .915–.917 for hypertension, .942–.942 for diabetes, and .740–.742 for dyslipidemia; SVM (the range from the hinge and squared hinge loss specifications), .916–.923, .944–.945, and .738–.748; neural network (the range from three different hidden units), .918–.922, .938–.940, and .748–.758.

The kNN with standardized input variables and logistic ridge failed to reach AUCs that were comparable to those of the logistic regression: kNN with standardized input variables (the range from the vote and IDW), .845–.847 for hypertension, .884–.885 for diabetes, and .675–.678 for dyslipidemia; logistic ridge, .892, .928, and .726.

The model which achieved the highest AUC for all three conditions, the ISLE with 1 for the subsampling ratio and 0.05 for the learning rate, yielded the following association measures at the representative coordinate on the ROC curve: hypertension, sensitivity 80.5%, specificity 95.8%, PPV 86.8%, and NPV 93.5%; diabetes, 89.8%, 94.7%, 60.5%, and 99.0%; dyslipidemia, 51.2%, 88.9%, 74.5%, and 74.2%.

## Discussion

Using health check-up results as the source of the gold standard, we demonstrated the association measures of the CBAs derived from machine learning methods without a condition-specific variable selection for identifying patients with three common chronic medical conditions, hypertension, diabetes, and dyslipidemia. This is the first study to investigate the benefits of machine learning methods in building CBAs comprehensively.

Among the logistic regression and penalized logistic regression, the logistic lasso and logistic elastic-net achieved the highest AUC, followed by logistic regression and logistic ridge. They are all linear in the parameter model with the same loss function, log-loss, but different penalty functions: zero penalties for logistic regression; an *L*_2_-penalty for logistic ridge; an *L*_1_-penalty for logistic lasso; and an elastic-net penalty for logistic elastic-net.

The methods using the *L*_1_-penalty are better suited to sparse and high-dimensional situations than those using zero penalties or the *L*_2_-penalty because of the selection of the effective input variables. These results are backed by theoretical results that support the superiority of the estimation methods that use the *L*_1_-penalty in sparse and high-dimensional settings [77–79]. Despite the fact that the prediction performance of the lasso is expected to be improved by the elastic-net if there is a group of variables among which the pairwise correlations are very high [46] and usually the diagnostic and medication codes corresponding to the target disease are highly correlated, we could not boost the AUC by the elastic-net compared with the lasso.

The tree-based model and neural network automatically select the input variables that are crucial to the discrimination and flexibly incorporate nonlinearity and interactions of them. The tree-based model largely attained superior AUC to any models and was at least as good as the benchmark cases. Among the tree-based models, the ISLE performed better than the random forest. Past Monte Carlo simulation studies have shown the superior performance of the ISLE to the random forest that uses the lasso post-processing in the aggregation process and the superior performance of the latter to the usual random forest [59, 60]. Therefore, two components of the ISLE are contributing to its superior performance to that of the random forest: learning term in the basis function generating process and lasso post-processing. The difference of the hyperparameter within the ISLE was not so much affecting the results.

In contrast to the tree-based model, the AUC of the neural network was not that high but comparable to that of the logistic regression. The performance of the neural network was much lower in the preliminary investigation that used a smaller sample size. The number of parameters in the neural network is nearly 7500, 15000, and 30000 for 5, 10, and 20 hidden units, respectively. Although the use of weight decay should alleviate the overfitting of the parameters to some extent, the sample size may still be insufficient for the neural network to demonstrate its true predictive power. As using multiple hidden layers with constraints such as local connectivity and weight sharing on the network, which allow for more complex connectivity but fewer parameters, improved the performance of the neural network dramatically in the field of image recognition [80, 81], it may also improve the performance of the neural network in the current subject. Increasing the sample size of data and devising more complex connectivity that suits the situation are fruitful directions for future research.

The AUC of the kNN with raw input variables was as good as that of the logistic regression, but that of the kNN with standardized input variables was lower. As is implicated by the difference of the AUC of the kNN with raw and standardized input variables, designing the distance metric in the kNN is difficult. If the input variables are standardized, the model is coerced to attach less importance on the input variables with high standard deviation, such as age and gender, than otherwise. Although the kNN had established an era in image recognition by the invention of the tangent distance [82], there is no such versatile distance measure yet in the field of CBA or studies using administrative data. It may be possible to improve the performance of the kNN by applying an unsupervised learning method that extracts essential components of the input variables, for example, principal component analysis [83], before measuring the distance. Although we do not probe further in this study, this is one direction of future research.

The AUC of the SVM was higher than that of the logistic ridge. They are linear in the parameter model with the same *L*_2_-penalty but different loss functions. The logistic ridge uses the log-loss, while the SVM uses the (squared) hinge loss. The hinge losses give zero penalties to points correctly classified and outside the margin. On the other hand, the log-loss gives continuously decreasing penalties as the correctly classified points get farther from the boundary of the margin. This feature of the hinge losses makes the SVM more robust to outliers than the other methods that are using the log-loss. Since most of the enrollees were far from the margin or outliers (i.e., most of them could be easily labeled as disease or non-disease by the CBA), the SVM is achieving a higher performance by better-discriminating enrollees with and without the target disease near the boundary than the other methods.

By comparing the results from the two datasets prepared for the logistic regression, we can see that the AUC declined for diabetes without a condition-specific variable selection. The inconsistency of the trend of the AUC among the target conditions demonstrates the trade-off between the accuracy and variance of the prediction function. When the number of the input variables of the prediction model becomes large relative to the sample size, there is a potential accuracy gain from the use of rich information and a possibility of a variance increase due to the variance inflation of the parameter estimates. In diabetes, the main dataset did not provide enough accuracy gain to offset the variance inflation, as the sample size was relatively small and the factors of being diagnosed as the target condition are successfully captured in the condition-specific variable selection (i.e., a high AUC is achieved by the alternative dataset). Conversely, in dyslipidemia, since the factors of being diagnosed as the condition seem to be not sufficiently covered by the condition-specific variable selection, the effect of the accuracy gain outweighs that of the variance increase.

There are potentially various ways of refining the AUC obtained in this study, drawing on the context of machine learning. Although the objective of this study is not to seek high AUC or prediction accuracy but to outline the prospect of the development of an efficient CBA construction procedure, we briefly introduce the concepts that are expected to become significant in the future accuracy pursuit of CBAs. The first one is more complicated and sophisticated learning models flourished in the field of machine learning, such as deep learning models [84]. The second is the pre-processing techniques that transform datasets *ex-ante* to utilize the power of learning machines more efficiently. There are mainly two approaches for pre-processing: methods that deal with imbalanced datasets [85] and those that perform feature selection [86]. The last one is error analysis in the performance analysis and debugging step of model building [87]. How one can successfully use these methods in CBA or, more broadly, claims data situation should be a worthwhile subject to be pursued.

We note that, admittedly, the most demanding and time-consuming task when conducting CBA research will usually be the construction of gold standards. For instance, most previous studies reviewed medical charts to construct the gold standard [13, 15, 16, 18, 19, 21–23, 25, 26, 28, 36, 39, 41]. Nevertheless, we still believe that our proposed method may also lower the bar of CBA research and useful for the following three reasons. Firstly, the performance measures calculated using appropriate machine learning methods can be potentially useful as a reference point, even when creating CBAs manually or exploring new CBA construction procedures.

Secondly, in some cases, it may be possible to sidestep the burden of chart reviewing by using regularly collected data like annual health screening results, which are used in this study. Electronic medical records and disease registries are possible candidates along this line. An increasing number of phenotype algorithms [88–90] may well function as gold standards for CBA research when electronic medical records are available. Cancer registries can be used to conduct comprehensive CBA research for various cancers. In fact, some CBA research is using health screening results [27, 33], blood test results from electronic medical records [22, 31], and disease registries [9, 14, 17, 24, 29, 40]. If this is the case, researchers can construct the gold standards from the regularly collected data without a serious burden and may be able to apply our proposed method to construct CBAs for a broad set of target conditions once an initial set of input variables are selected.

Finally, as we highlighted in the Introduction, there are demands to build CBAs for a wide variety of diseases efficiently. For instance, we need to renew CBAs when the coding scheme changes [42, 43], and a number of countries are still suffering from a lack of CBAs [44]. The lack of confirmed CBAs degrades the research quality, and the research will likely fail to attract high impact factor journals’ attention [45].

There is a two-dimensional generalizability issue on the value of association measures computed here: the study population only covers regular employees; the research only dealt with three conditions, hypertension, diabetes, and dyslipidemia. Additionally, input variables selected without using subject-matter knowledge on the target conditions in this study may be inadequate for other situations and conditions. Additional enrollee characteristics, ICD-10/WHO-ATC codes with three or more digits, and procedure codes may need to be included to attain satisfactory CBAs. The information on primary diagnoses and suspected cases may also be helpful. However, considering that comorbidities were our focus, we do not expect that incorporating those types of information would appreciably affect the methods’ accuracy in this study. Lastly, the learning method that suits may depend on the target condition. We hope that similar studies will be conducted on situations other than those that were investigated in the present research to gain a deeper understanding regarding the development of efficient CBA research.

In sum, the penalized logistic regressions other than ridge and tree-based models, which are the leading machine learning methods, achieved AUCs comparable to the logistic regression with a knowledge-based condition-specific variable selection. Besides, the AUC level was satisfactory for hypertension and diabetes. Appropriate machine learning methods can substitute our knowledge of target conditions to construct CBAs efficiently.

## Supporting information

S1 File Receiver operating characteristic curve for claims-based algorithms derived from machine learning methods (A, Hypertension; B, Diabetes; C, Dyslipidemia). *Abbreviations*: AUC, area under the receiver operating characteristic curve; IDW, inverse distance weighting; ISLE, importance sampled learning ensemble; kNN, k-nearest neighbor; Std., standardized; SVM, support vector machine; RF, random forest. Notes: Age, gender, and all International Classification of Diseases and Related Health Problems, Tenth Revision (ICD-10)/World Health Organization-Anatomical Therapeutic Chemical (WHO-ATC) codes with a letter followed by two digits were used as input variables for all models but the logistic regression using the alternative dataset. The main logistic regression fitted a logistic regression model to the dataset that was appropriately trimmed. The Euclidean distance with raw or standardized (i.e., rescaled to have mean zero and variance one) input variables was adopted as a distance metric for the k-nearest neighbor (kNN). The number of the nearest neighbors to be counted, k, was optimized using the validation set. The predicted class probabilities that were computed from (1) the frequency of the class of the k-nearest neighbors (vote) and (2) the inverse distance weighted frequency of the class of the k-nearest neighbors (IDW) composed a prediction function. A linear basis function with a hinge or squared hinge loss was adopted in the support vector machine (SVM). The cost parameter was optimized using the validation set. Decision values (i.e., the distance of the point from the hyperplane) made up a prediction function. From the penalized regression, logistic regression with the L_2_-penalty (logistic ridge), L_1_-penalty (logistic lasso), and elastic-net penalty (logistic elastic-net) were applied. The regularization coefficient and elastic-net mixing parameter were determined by cross-validation. Two types of tree-based models were applied: random forest and importance sampled learning ensemble (ISLE). The minimum node size was set to 10 for each tree, and 200 trees were bagged in the random forest. The number of variables selected for each split was tuned using the validation set. We fixed the depth to be six for the ISLEs. As the combination of the subsampling ratio for each tree and the learning rate, we selected (1,0.05), (0.5,0.1), and (0.1,0.1). The number of trees and regularization coefficient were determined by cross-validation. The L_1_-penalty was adopted in the post-processing. A single hidden layer neural network was applied with a different number of hidden units: 5, 10, and 20. All hidden units were fully connected with the nodes in the input and output layers. Weight decay was employed for the regularization of parameters, and the regularization coefficient of it was tuned using the validation set.
(DOCX)
