# Supplementary material for: Claims-based algorithms for common chronic conditions were efficiently constructed using machine learning methods
Source: PLoS One. 2021 Sep 27;16(9):e0254394. doi: 10.1371/journal.pone.0254394 (PMC8476042; doi:10.1371/journal.pone.0254394)
Supplement: S1 File — Abbreviations: AUC, area under the receiver operating characteristic curve; IDW, inverse distance weighting; ISLE, importance sampled learning ensemble; kNN, k-nearest neighbor; Std., standardized; SVM, support vector machine; RF, random forest. Notes: Age, gender, and all International Classification of Diseases and Related Health Problems, Tenth Revision (ICD-10)/World Health Organization-Anatomical Therapeutic Chemical (WHO-ATC) codes with a letter followed by two digits were used as input variables for all models but the logistic regression using the alternative dataset. The main logistic regression fitted a logistic regression model to the dataset that was appropriately trimmed. The Euclidean distance with raw or standardized (i.e., rescaled to have mean zero and variance one) input variables was adopted as a distance metric for the k-nearest neighbor (kNN). The number of the nearest neighbors to be counted, k, was optimized using the validation set. The predicted class probabilities that were computed from (1) the frequency of the class of the k-nearest neighbors (vote) and (2) the inverse distance weighted frequency of the class of the k-nearest neighbors (IDW) composed a prediction function. A linear basis function with a hinge or squared hinge loss was adopted in the support vector machine (SVM). The cost parameter was optimized using the validation set. Decision values (i.e., the distance of the point from the hyperplane) made up a prediction function. From the penalized regression, logistic regression with the L2-penalty (logistic ridge), L1-penalty (logistic lasso), and elastic-net penalty (logistic elastic-net) were applied. The regularization coefficient and elastic-net mixing parameter were determined by cross-validation. Two types of tree-based models were applied: random forest and importance sampled learning ensemble (ISLE). The minimum node size was set to 10 for each tree, and 200 trees were bagged in the random forest. The number of variables se [file pone.0254394.s001.docx]

**A. Hypertension**

**B. Diabetes**

**C. Dyslipidemia**
